# Case report: A 9-year systematic treatment failure of a pulmonary tuberculosis patient

**DOI:** 10.3389/fpubh.2022.966891

**Published:** 2022-09-06

**Authors:** Hui Jiang, Chendi Zhu, Liyi Qin, Xiaoguang Wu, Jinfeng Yin, Yijia Guo, Huan Ma, Junnan Jia, Fengmin Huo, Yi Xue, Mengqiu Gao, Weimin Li

**Affiliations:** ^1^Beijing Chest Hospital, Capital Medical University, Beijing, China; ^2^Beijing Tuberculosis and Thoracic Tumor Research Institute, Beijing, China; ^3^National Tuberculosis Clinical Laboratory, Beijing Tuberculosis and Thoracic Tumor Research Institute, Beijing, China; ^4^Beijing Key Laboratory for Drug Resistant Tuberculosis, Beijing, China; ^5^Department of Tuberculosis, Beijing Chest Hospital, Capital Medical University, Beijing, China; ^6^School of Public Health, Wenzhou Medical University, Wenzhou, China

**Keywords:** tuberculosis, treatment failure, whole genome sequencing, multidrug resistance, computed tomography-three dimensional

## Abstract

**Objective:**

To explore the reasons of failure in a case of pulmonary tuberculosis (PTB) after 9 years systematic treatment.

**Methods:**

We extracted the patients' treatment history, drug susceptibility testing (DST), Computed tomography (CT) images, and sequenced the isolated strains by whole gene sequencing (WGS).

**Results:**

Although most results of the phenotypical DSTs were consistent with the genotype DST, the occurrence of gene resistance to amikacin (AMK), capreomycin (CAP), moxifloxacin (MFX) was earlier than the phenotypical DST. Based on the continuously reversed results of phenotypical DSTs, CT images in different stages and WGS, it can be confirmed that the patient was infected with two different strains of Mycobacterium tuberculosis (M.TB). Moreover, severe cavities may be another factor leading to treatment failure.

**Conclusion:**

Given the suggestive effect of genotype DST is earlier than the phenotypical DST, so genotype DST can play a better guiding role in patients with MDR-TB. Additionally, for patients who have not been cured for a long time, medication should be more cautious and the role of WGS in drug resistance surveillance should be fully utilized.

We report and discuss a case of pulmonary tuberculosis (PTB) with failure after 9 years (February 2012, to July 2021) systematic treatment. The immunodeficiency workup on the patient, a 29-year-old female, revealed normal immunoglobulin levels and a negative result for the anti-HIV human T-lymphotropic virus. In addition, further relevant analysis showed no evidence of other comorbidities including hepatitis, diabetes, and other respiratory diseases. By analyzing her treatment history, drug susceptibility testing (DST), Computed tomography (CT) images, and isolated strains' whole genome sequencing (WGS), we hope to identify the causes of treatment failure and seek expert guidance to improve her survival time and quality of life (A detailed case report is provided in the Supplementary Appendix).

The case's whole treatment history can be divided into three stages: in the first stage (February 2012 to March 19, 2017), the patient was hospitalized in the local tuberculosis-designated hospital in February 2012 with her first pulmonary tuberculosis diagnosis and began to receive first-line antituberculosis regimen. After 1 year of treatment, she decided to stop treatment with improvement in March 2013. Only 2 months later, she was admitted to Beijing Chest Hospital due to the recurrence and deterioration of the previous clinical manifestations and then treated with para-aminosalicylate (PAS), rifabutin (RFB), pyrazinamide (PZA), amikacin (AMK), ethambutol (EMB), and levofloxacin (LFX) for 2 years but not cured. On March 19, 2017, CT images showed the lesions increased and progressed. Throughout this stage of therapy, DSTs results were absent. In the second stage (March 20, 2017, to June 12, 2019), the patient received standardized treatment for multidrug-resistant tuberculosis (MDR-TB) under DST results and was not cured. In this stage, based on the DST results, the patient was treated with TB sensitive drugs, including cycloserine (CS), linezolid (LZD), capreomycin (CM), moxifloxacin (MFX), clofazimine (CFZ), but the deterioration continued to progress. In addition, the CT images showed that two pre-existing independent thick-walled cavities 1 and 2 in the right upper lung had partial absorption. In the third stage (June 13, 2019, to July 23, 2021), the patient received Bedaquiline (BDQ) and Delamanid (DLM) combinated with the previous standardized treatment but was still not cured. In the absence of accessible therapeutic medications and after declining our surgical recommendations, the patient was gradually transitioned to palliative care. Interestingly, the DSTs results on June 13, 2019, and August 24, 2020, showed that LFX, protionamide (PTO) and isoniazid aminosalicylate (PA) changed from drug resistance to sensitivity. However, the subsequent five phenotypical DSTs results from October 30, 2020, to July 23, 2021, were similar to those in 2017. In addition, CT images presented those two cavities fused from connected locally to extensively. The fusion lesion infiltrated outward and deteriorated rapidly, resulting in the destruction of the entire right upper lobe (Figures 1A,B, Table 1).

**Figure 1:**
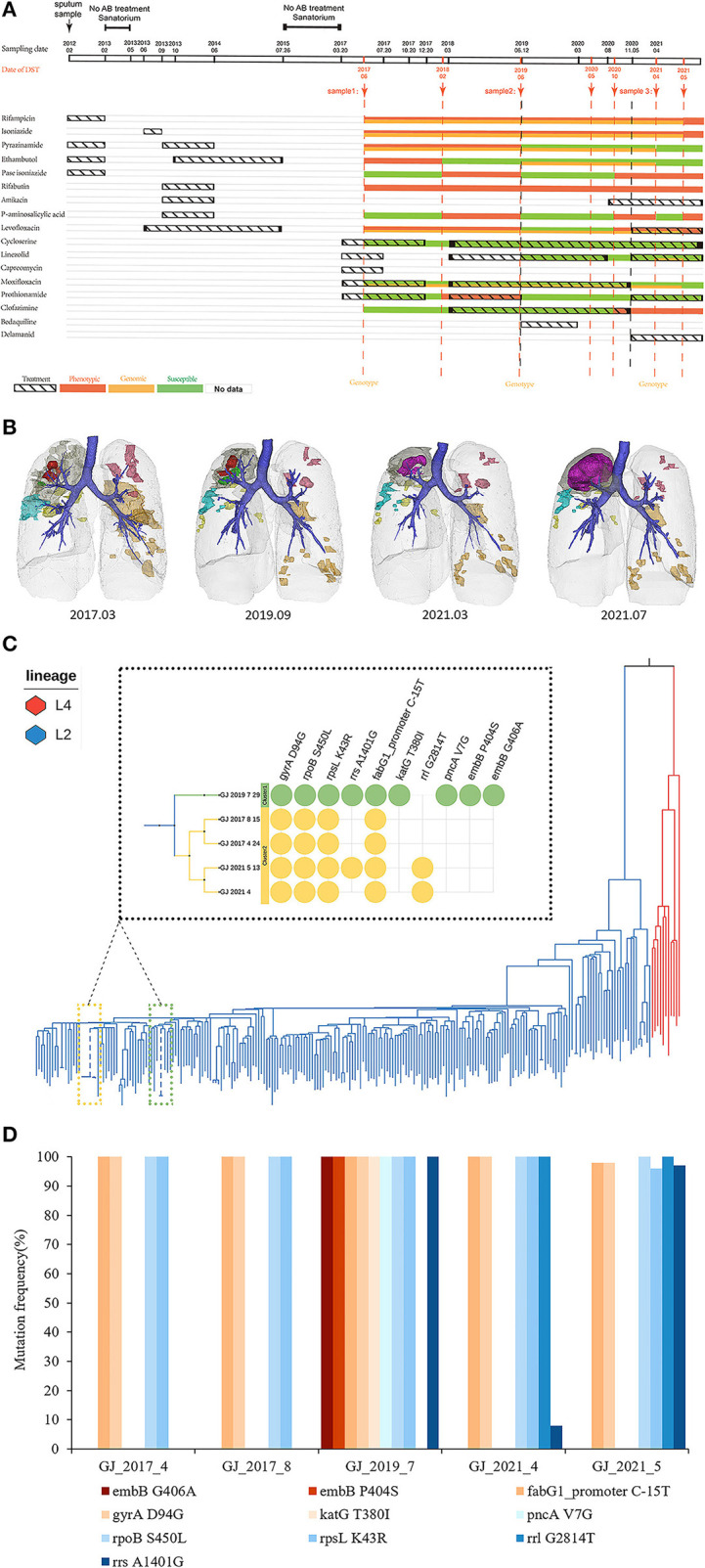
Treatment history, CT images, phylogenetics and drug resistance profiles, and unfixed mutation subgroups of drug resistance genes. **(A)** Treatment History. Anti-tuberculosis drugs used in different periods. **(B)** Three-dimensional visualization of CT scans demonstrates the longitudinal anatomical change in lung lesions. Lesions in different lung lobes are shown in different colors, and the trachea (blue) and pulmonary contours (light gray) are shown as references. There were two independent cavities 1 (red) and 2 (green) in the right upper lobe parenchymal abnormality (dark gray) in March 2017, which appeared partial absorption in September 2019, and subsequently fused extensively and infiltrated outward (magenta) from March to July 2021. Such rapid deterioration resulted in the destruction of the right upper lung lobe. All other lung lobar lesions showed a benign response to treatment or remained relatively stable. Lesions of the right middle lobe (cyan), right lower. lobe (yellow), left upper lobe (pink), and left lower lobe (orange) are also shown in the **(B)**. **(C)** Whole-genome sequencing. Five strains belong to L2. Mutations that confer drug resistance are shown. FLUOROQUINOLONES (*gyrA*), RIFAMPICIN (*rpoB*), STREPTOMYCIN (*rpsL*), AMIKACIN (*rrs*), LINEZOLID (*rrl*), ISONIAZID (*fabG1_promoter, katG*), PYRAZINAMIDE (*pncA*), ETHAMBUTOL (*embB*). **(D)** Unfixed mutation subgroups of drug resistance genes. Resistance to amikacin developed in 2021. Percentages on the y axis are based on the number of genome-sequencing reads supporting the corresponding drug resistance-conferring mutation.

**Table 1:** The results of drug susceptibility test to 16 drugs from 2017 to 2021.

**Date**	**RFP**	**INH**	**EMB**	**RFB**	**SM**	**AMK**	**PAS**	**LFX**	**CPM**	**MFX**	**PTO**	**CFZ**	**RFT**	**PI**	**CLR**	**KAN**
2017.3.28	R	S	S	R	R	S	S	R	S	S	S	S	R	S	S	S
2017.4.13	R	S	S	R	R	S	S	R	S	S	S	S	R	S	S	S
2017.4.14	R	S	S	R	R	S	S	R	S	S	S	S	R	S	S	S
2017.4.18	R	S	S	R	R	S	S	R	S	S	S	S	R	S	S	S
2017.6.29	R	S	S	R	R	S	S	R	S	S	S	S	R	R	S	S
2017.6.30	R	S	S	R	R	S	S	R	S	S	S	S	R	R	S	S
2017.7.03	R	S	S	R	R	S	S	R	S	S	S	S	R	R	S	S
2018.2.8	R	R	S	R	R	S	R	R	S	S	R	S	R	R	S	S
2018.3.15	R	R	S	R	R	S	S	R	S	S	R	S	R	R	S	S
2019.6.13	R	R	S	R	R	S	S	S	S	S	S	S	R	S	S	S
2020.8.24	R	R	S	R	R	S	S	S	S	S	S	S	R	S	S	S
2020.10.30	R	R	S	R	R	S	R	R	S	S	S	R	R	R	R	R
2021.03.26	R	R	S	R	R	S	S	R	S	S	S	R	R	R	S	R
2021.03.26	R	R	S	R	R	S	S	R	S	S	S	R	R	R	S	R
2021.04.23	R	R	S	R	R	S	S	R	S	S	S	R	R	R	S	R
2021.07.23	R	R	S	R	R	R	R	R	R	S	S	R	R	R	S	R

RFP, rifampicin; INH, soniazid; EMB, ethambutol; RFB, rifabutin; SM, streptomycin; AMK, amikacin; PAS, para-aminosalicylate; LFX, levofloxacin; CPM, capreomycin; MFX, moxifloxacin; PTO, protionamide; CFZ, clofazimine; RFT, rifapentine; PI, isoniazid-aminosalicylate; CLR, clarithromycin; KAN, kanamycin; R, resistance; S, susceptible.

Based on the continuously reversed results of phenotypical DSTs results and CT images in different stages, we speculate that the patient was infected with two different strains of *Mycobacterium tuberculosis* (*Mtb*). To verify our conjecture, the five existing sputum samples sampled in Apr 2017, Aug 2017, Jun 2019, Apr 2021, and May 2021 were sequenced by the whole genome (Details of WGS and Variant calling are provided in the Supplementary Appendix). All five strains belonged to lineage 2.3, differing by up to 196 SNPs, of which the strain in 2019 is a separate isolate (cluster 1), and the remaining four strains are from the same cluster (cluster 2). In view of the diversity of strains *in vivo* hardly exceeds 12 SNPs, (1) we speculate that this patient has mixed infection. The two clusters shared four mutations in *rpoB, fabG1_promoter, rpsL* and *gyrA*, which were associated with rifampicin (RFP), isoniazid (INH), streptomycin (SM) and fluoroquinolones resistance, respectively. Additionally, five independent mutations in cluster 1 were associated with first-line drug resistance (INH, EMB, PZA), consistent with the patient's first-stage treatment history. Given that drug selection pressure is the primary driver of resistance mutations in strains, (2) and no EMB and PZA related mutations were found in cluster 2, we speculate that cluster 1 was the first infected strain of this patient (Figures 1C,D). This was reflected in the emergence of mutations in *rrl*. Therefore, we speculated that the two strains coexisted in the lung for a long time, resulting in the failure of the patient's treatment.

Summarizing the above information, although most results of the phenotypical DSTs results were consistent with the genotype DST results, the occurrence of gene resistance to AMK, capreomycin (CAP), MFX was earlier than the phenotypical DST results, which indicates that the drug resistance mutations had gradually accumulated during the treatment, but the phenotypical DST results was not prompted in time. The treatment scheme was not changed in time, resulting in the gradual accumulation of resistance and treatment failure. In addition, with the advent of new drugs such as BDQ and DLM, the cure rate of multidrug-resistant TB can reach over 80% (3). However, this patient still failed to respond to treatment without resistance to BDQ and DLM, which may be related to severe cavities (4) The necrotic tissue in the cavities is not only the rich medium of *Mtb*, (5) but also the cavity walls composed of connective tissue may hinder the drugs' penetration, (6) which is easier to induce *Mtb* to produce resilience, tolerance and resistance.

This study has some limitations. First, the treatment failure in the first stage can be explained from the genotype resistance results of the strain in April 2017, but we have no way of knowing the resistance situation and evolution process of the strains before. Second, the early strains were not preserved during the 9-year treatment process, so the sequencing of infection of the two clusters cannot be accurately obtained and the cumulative changes of drug resistance genes could not be dynamically observed.

In this study, we found that genotype DST findings, which are sooner than phenotypical DST results, can provide superior treatment guidance for tuberculosis. In addition, mixed infection of *Mtb* leads to the emergence of heterogeneous flora, making the treatment more complex. Therefore, we should be more cautious when prescribing medication to patients who haven't been healed for a long time and utilize WGS for drug resistance surveillance.

## Data availability statement

The datasets presented in this study can be found in online repositories. The names of the repository/repositories and accession number(s) can be found at: https://www.ncbi.nlm.nih.gov/bioproject/, PRJNA868171.

## Ethics statement

The studies involving human participants were reviewed and approved by Ethics Committee of Beijing Chest Hospital, Capital Medical University. The patients/participants provided their written informed consent to participate in this study.

## Author contributions

WL, MG, and HJ conceived, designed, and supervised the study. XW, JJ, FH, YX, and YG collected the data. CZ and JY analyzed WGS data. LQ analyzed CT data. CZ, JY, LQ, YG, and HM created the figures. HJ, CZ, LQ, and XW interpreted the findings. HJ wrote the drafts of the manuscript. WL and MG commented on and revised the drafts of the manuscript. All authors read and approved the final manuscript.

## Funding

This study was funded by grants from Beijing Key Clinical Specialty Project (20201214) and the National Natural Science Foundation of China (U1903118). The funder of the study had no role in the study design, data collection, data analysis, data interpretation, writing of the report, or the decision to publish.

## Conflict of interest

The authors declare that the research was conducted in the absence of any commercial or financial relationships that could be construed as a potential conflict of interest.

## Publisher's note

All claims expressed in this article are solely those of the authors and do not necessarily represent those of their affiliated organizations, or those of the publisher, the editors and the reviewers. Any product that may be evaluated in this article, or claim that may be made by its manufacturer, is not guaranteed or endorsed by the publisher.

## References

[1] Liu Q Wei J Li Y Wang M Su J Lu Y. Mycobacterium tuberculosis clinical isolates carry mutational signatures of host immune environments. Sci Adv. (2020) 6:eaba4901. 10.1126/sciadv.aba4901 32524000 PMC7259932

[2] Nguyen QH Contamin L Nguyen TVA Bañuls AL. Insights into the processes that drive the evolution of drug resistance in *Mycobacterium tuberculosis*. Evol Appl. (2018) 11:1498–511. 10.1111/eva.12654 30344622 PMC6183457

[3] Liu Y Gao M Du J Wang L Gao J Shu W. Reduced susceptibility of *Mycobacterium tuberculosis* to Bedaquiline during antituberculosis treatment and its correlation with clinical outcomes in China. Clin Infect Dis. (2021) 73:e3391–e7. 10.1093/cid/ciaa1002 32667984

[4] Urbanowski ME Ordonez AA Ruiz-Bedoya CA Jain SK Bishai WR. Cavitary tuberculosis: the gateway of disease transmission. Lancet Infect Dis. (2020) 20:e117–28. 10.1016/S1473-3099(20)30148-1 32482293 PMC7357333

[5] Sarathy JP Dartois V. Caseum: a niche for *Mycobacterium tuberculosis* drug-tolerant persisters. Clin Microbiol Rev. (2020) 33:e00159–19. 10.1128/CMR.00159-19 32238365 PMC7117546

[6] Strydom N Gupta SV Fox WS Via LE Bang H Lee M. Tuberculosis drugs' distribution and emergence of resistance in patient's lung lesions: a mechanistic model and tool for regimen and dose optimization. PLoS Med. (2019) 16:e1002773. 10.1371/journal.pmed.1002773 30939136 PMC6445413

