# Supplementary material for: Case report: A 9-year systematic treatment failure of a pulmonary tuberculosis patient
Source: Front Public Health. 2022 Sep 6;10:966891. doi: 10.3389/fpubh.2022.966891 (PMC9487995; doi:10.3389/fpubh.2022.966891)
Supplement: Supplementary file 1 [file Data_Sheet_1.pdf]

SUPPLEMENTAL APPENDIX:

Case presentation：

Here we present a 9-year history of systematic treatment failure in a 29-year-old female with pulmonary tuberculosis (PTB). Her immunodeficiency workup revealed normal immunoglobulin levels and a negative result for the anti-HIV human T-lymphotropic virus. In addition, further relevant analysis showed no evidence of other comorbidities including hepatitis, diabetes, and other respiratory diseases. The entire anti-tuberculosis treatment history can be divided into three stages, as detailed in the following case report.

Stage I (02/2012-03/2017)

With cough, sputum production, bilateral lung shadows on chest radiograph, and acid-fast bacillus-positive (AFB+) sputum microscopy, the patient was hospitalized in the local tuberculosis-designated hospital in February 2012 and first given the diagnosis of pulmonary tuberculosis. She began to receive first-line antituberculosis regimen (Rifampicin, Isoniazid, Ethambutol and Pyrazinamide). After one year of treatment, the patient decided to stop treatment with improvement in March 2013.

Only two months later, that is, in May 2013, as the recurrence and deterioration of the previous clinical manifestations, the patient was admitted to Beijing Chest Hospital and re-treated with para-aminosalicylate (PAS), rifabutin (RFB), pyrazinamide (PZA), amikacin (AMK), ethambutol (EMB), and levofloxacin (LFX). After two years of treatment, the patient voluntarily terminated the treatment and announced the loss of follow-up in May 2015.

After 18 months later, the patient recurred. In the next four months, the patient took some antibiotics (unknown) and cough suppressants by herself. In view of no improvement, she was subsequently re-registered in March 2017.

Stage II (03/2017-06/2019)

After re-registered on 20/03/2017, the patient was diagnosed with Rifampicin resistance using drug susceptibility testing (DST). Baseline Computed Tomography (CT) showed consolidation in the right upper lobe and bilateral cavitation. Results from her initial sputum specimens were 3+ for AFB smear microscopy. On 17/04/2017, she began to receive a standardized regimen, including cycloserine (CS), linezolid (LZD), capreomycin (CM), and moxifloxacin (MFX). In the fifth month (08/2017), CM was withdrawn due to the adverse effects of electrolyte disturbance. With the addition of PAS, the treatment was modified to include PAS, CS, MFX, and LZD. Follow-up CT taken three months and seven months after treatment initiation showed absorption of lesions. However, sputum specimens collected at month eight were still smear- and culture-positive.

At month ten, the patient was recommended to change the regimen because of the lack of absorption of lung lesions and continuous bacterial positive results, which was refused until the month twelve (13/03/2018). Subsequently, clofazimine (CFZ) was added to the previous regimen to strengthen the efficacy of the therapy. She received this revised regimen from March 2018 to June 2019. During this 15-month treatment, follow-up routine CT showed that lesions in the left lung continued to improve, but only partially in the right lung, and more importantly, consolidation and cavitation in the right upper lobe deteriorated at 12 months following new regimen initiation. The patient remained culture-positive late in this regimen.

Stage III (06/2019-07/2021)

Between June 2019 and March 2020, the patient received Bedaquiline (BDQ) on a compassionate basis for 9 months, and other background drugs including PAS, CS, MFX, LZD and CFZ continued until November 2020. Although the addition of BDQ reversed previous deterioration of lesions, the situation rebounded with the background drugs alone. Interestingly, the DSTs results on 13/06/2019 and 24/08/2020, showed that LFX, protionamide (PTO) and isoniazid aminosalicylate (PA) changed from drug resistance to sensitivity, which is different from the previous resistance pattern.

On November 15, 2020, Delamanid (DLM) was added to the previous standardized treatment. The phenotypic DSTs results thereafter until July 2021 were similar to those in 2017. The lesions, especially those in the right upper lobe, did not show significant improvement during the initial course of treatment, but instead deteriorated rapidly from May to July 2021. CT images also revealed that cavities in the right upper lobe gradually merged and infiltrated outward, resulting in the destruction of the entire lobe. The patient was classified as treatment failure and was gradually transitioned to palliative care.

WGS and Variant calling:

A 300-base pair fragment length library was constructed for each DNA sample, and WGS was performed on an Illumina HiSeq 2500 system with either the single-end or paired-end strategy. We used a previously validated pipeline for mapping short sequencing reads to the reference genome[1] The Sickle[2] tool was used to trim the WGS data and sequencing reads with a Phred base quality above 20 and read length longer than 30 were kept for analysis. Bowtie 2 (version 2.2.9) [3]was used to map sequencing reads to M. tuberculosis H37Rv strain (NC_000962.2) as a reference template. SAMtools (version 1.3.1)[4] was used for SNP calling with mapping quality greater than 30 and fixed mutations (frequency ≥75%) were identified using VarScan (version 2.3.9)[5]. We discarded low-quality isolates with an average sequencing depth lower than 20. We excluded SNPs in repetitive regions of the genome (PPE/PE-PGRS family genes, phage sequences, insertions, or mobile genetic elements) and identified small INDELs.

Files containing sequencing reads were deposited in the National Institutes of Health Sequence Read Archive (BioProject PRJNA868171).

Reference

1. Liu Q, Ma A, Wei L, et al. China's tuberculosis epidemic stems from historical expansion of four strains of Mycobacterium tuberculosis. *Nat Ecol Evol*. 2018 Dec;2(12):1982-1992.
2. Joshi NA, Fass JN. (2011). Sickle: A sliding-window, adaptive, quality-based trimming tool for FastQ files (Version 1.33) [Software]. Available at https://github.com/najoshi/sickle.
3. Langmead B, Salzberg SL. Fast gapped-read alignment with Bowtie 2. *Nat Methods*. 2012 Mar 4;9(4):357-9.
4. Li H, Handsaker B, Wysoker A, et al. The Sequence Alignment/Map format and SAMtools. *Bioinformatics.* 2009 Aug 15;25(16):2078-9.
5. Koboldt DC, Zhang Q, Larson DE, et al. VarScan 2: somatic mutation and copy number alteration discovery in cancer by exome sequencing. *Genome Res.* 2012 Mar;22(3):568-76.
